# Mulberry-based bio-reclamation of salinity-degraded landscapes

**DOI:** 10.1016/j.isci.2026.116566

**Published:** 2026-06-30

**Authors:** Ritwik Acharya, S. Rehan Ahmad, Debnirmalya Gangopadhyay, Shubhajit Shaw, Ankita Manna, Sreya Das, Soumi Chakraborty, Debasmita Sen, Rahul Chatterjee

**Affiliations:** 1 Department of Botany, Hiralal Mazumdar Memorial College for Women, Kolkata 700035, West Bengal, India; 2 Department of Zoology, Hiralal Mazumdar Memorial College for Women, Kolkata 700035, West Bengal, India; 3 Department of Sericulture, Raiganj University, Raiganj 733134, West Bengal, India; 4 Department of Botany, University of Calcutta, Kolkata 700019, West Bengal, India; 5 Department of Environmental Science, Asutosh College, Kolkata 700026, West Bengal, India; 6 Department of Environmental Science, Indira Gandhi National Open University, New Delhi 110068, India

**Keywords:** soil salinization, mulberry, saline agriculture, bio-reclamation, environmental management, ecological restoration

## Abstract

Soil salinization affects more than 1 billion hectares globally and 6.74 million hectares in India, causing substantial productivity losses and degradation of soil ecological function. This review synthesizes evidence from the literature (1977–2025) to evaluate mulberry (*Morus* spp.) as a nature-based solution (NbS) for bio-reclamation of salinity-degraded landscapes within agroforestry restoration systems. Genotype-level analyses indicate mulberry tolerance to soil electrical conductivity (EC) up to 23 dS m^−1^, with operational EC thresholds of 4–8, 8–15, and 15–23 dS m^−1^. Mulberry enhances ion regulation, antioxidant defense and progressive soil structural recovery while supporting sericulture-linked livelihoods. An artificial intelligence (AI)-enabled decision-support system achieved strong predictive performance (coefficient of determination [R^2^] = 0.857; root-mean-square error [RMSE] = 0.769), illustrating how integration of ecological mechanisms and data-driven modeling can enable scalable, climate-resilient land restoration planning.

## Graphical abstract

**Figure:**
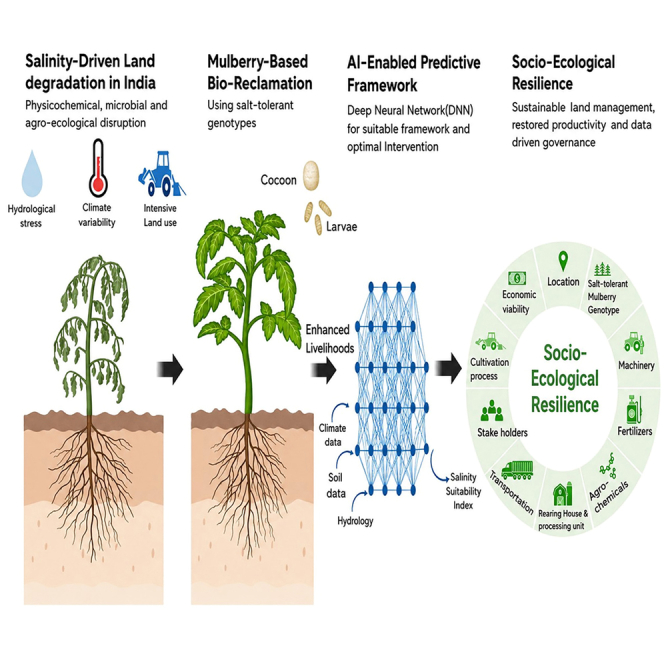

## Teaser

Ecology; environmental science; nature conservation

## Introduction

Soil salinization is among the most persistent forms of land degradation, affecting more than 1 billion hectares globally across arid, semi-arid, and coastal regions.^1^ Major hotspots mainly occur in major parts of Asia, the Mediterranean region, and Africa, where irrigation mismanagement, groundwater over-extraction, marine intrusion, and climate-driven hydrological shifts accelerate secondary salinization processes.^2^ In India alone, approximately 6.74 million hectares are affected, particularly within canal command systems and coastal states such as Gujarat and West Bengal.^3^

Salinity-induced degradation operates through coupled hydrogeochemical and biological pathways. Elevated soil electrical conductivity (EC > 4 dS m^−1^) and exchangeable sodium (Na^+^) destabilize soil aggregates, reduce hydraulic conductivity and restrict leaching.^4^^,^^5^ Excess Na^+^ and chloride (Cl^−^) disrupt plant ion homeostasis, impair photosynthesis and suppress nutrient uptake,^6^^,^^7^ while microbial biomass and enzymatic activity decline under increasing osmotic stress.^8^^,^^9^ These interacting processes result in yield losses of approximately 20%–50% in affected regions and progressively reduce soil resilience.^10^

Conventional reclamation strategies have largely relied on physicochemical and engineering interventions, particularly gypsum application and subsurface drainage.^11^^,^^12^ Although effective under favorable hydrological conditions, such measures are often capital-intensive due to the cost of chemical amendments and associated infrastructure.^13^ In contrast, biologically mediated and agroforestry-based approaches promote gradual structural stabilization, organic carbon accumulation and plant-soil feedback processes.^14^^,^^15^ However, these approaches remain insufficiently integrated into operational planning frameworks, and clearly defined salinity thresholds linked to plant performance are rarely established.^16^

Within this context, mulberry (*Morus* spp.) emerges as a multifunctional candidate for salinity bio-reclamation. Selected genotypes demonstrate sustained productivity at EC levels approaching 19 to 23 dS m^−1^^17^^,^^18^^,^^19^^,^^20^ Physiological tolerance mechanisms include selective Na^+^ exclusion, maintenance of favorable potassium-to-sodium (K^+^/Na^+^) ratios, and enhanced antioxidant defense.^21^^,^^22^ Beyond plant-level tolerance, mulberry’s perennial root architecture enhances soil structural stability and contributes to organic carbon accumulation,^23^ while integration with sericulture embeds ecological restoration within established livelihood systems.^24^^,^^25^

Nature-based solutions (NbSs) are defined as actions that protect, sustainably manage and restore natural or modified ecosystems while addressing environmental challenges and providing ecological and socio-economic benefits.^26^ Framed as an NbS, mulberry-based bio-reclamation relies on ecosystem processes including ion regulation, rhizosphere interactions, structural aggregation, and carbon inputs^23^ rather than solely external chemical amendments.^27^ By combining ecological recovery with socio-economic co-benefits, this approach aligns land restoration with sustainable rural development. Nevertheless, a potential scalable deployment requires clearly defined salinity thresholds, genotype-specific tolerance synthesis and a structured decision-support system linking soil indicators with suitability assessment.

Despite extensive literature on halophyte-assisted restoration and amendment-based reclamation,^14^^,^^15^^,^^28^ integration of mechanistic plant tolerance, operational EC thresholds, and predictive modeling systems remains limited. This review, therefore, synthesizes global and Indian evidence to examine soil and ecosystem responses to salinity, consolidate genotype-specific tolerance mechanisms in mulberry as NbS across EC gradients, define operational salinity thresholds linked to productivity and survival outcomes, and develop an artificial intelligence (AI)-enabled decision-support system integrating soil, climatic and genotype indicators. By linking ecological mechanisms with deployment criteria and predictive analytics, this study advances mulberry-based bio-reclamation toward scalable, data-supported land management planning.

## Novelty and contributions of this review

Previous reviews on salinity management have primarily focused on halophyte-assisted restoration systems, physicochemical reclamation strategies (e.g., gypsum application and drainage), or broad agroforestry-based rehabilitation models.^14^^,^^15^^,^^28^ In this review, mulberry-based bio-reclamation is conceptualized as a species-specific NbS embedded within agroforestry restoration systems, linking plant physiological tolerance with ecosystem-level recovery processes. While these syntheses provide valuable insights into vegetative stabilization and soil amendment approaches, they generally do not integrate genotype-specific physiological tolerance mechanisms with operational deployment frameworks. Furthermore, existing agroforestry-oriented reviews rarely establish clearly defined EC-based salinity thresholds linked to crop productivity, nor do they incorporate structured decision-support systems for site prioritization.

In contrast, the present review advances the literature in four specific and integrative dimensions. First, it centralizes mulberry as a primary genus for bio-reclamation rather than treating it as a peripheral example within broader agroforestry systems. Second, it synthesizes genotype-specific physiological and biochemical tolerance mechanisms across defined EC gradients, thereby linking mechanistic plant responses with salinity intensity. Third, it establishes operational ecological applicability thresholds (4–8, 8–15, and 15–23 dS m^−1^) explicitly connected to field productivity and survival outcomes. Finally, it proposes an AI-enabled decision-support system for site suitability selection incorporating a deep neural network (DNN) that integrates soil physicochemical properties, climatic variables and genotype-specific performance indicators to support structured, potentially scalable deployment of mulberry-based bio-reclamation.

By integrating ecological mechanisms, physiological evidence, livelihood-oriented deployment and a structured decision-support system within a unified analytical framework, this review moves beyond descriptive restoration narratives and provides an operational blueprint for scalable mulberry-based bio-reclamation of salinity-degraded landscapes.

## Methodology

This review followed a systematic evidence-synthesis approach guided by the preferred reporting items for systematic reviews and meta-analyses (PRISMA) framework to ensure transparency and reproducibility in the literature selection process. The objective was to synthesize ecological, physiological, and environmental management evidence related to soil salinity impacts and the potential of mulberry (*Morus* spp.) as a bio-reclamation strategy for salinity-degraded landscapes. Prior to the review, a protocol outlining the search strategy, eligibility criteria, and data extraction procedures was developed to maintain consistency in study identification and evaluation.

A comprehensive literature search was conducted across Scopus, Web of Science, ScienceDirect, PubMed, AGRIS, and Google Scholar, covering peer-reviewed publications from 1977 to 2025. Search terms were organized around four thematic domains: soil salinity and degradation processes, salinity management and ecological restoration approaches, mulberry salt tolerance and genotype evaluation, and emerging approaches for salinity monitoring and assessment. Reference lists of relevant articles and technical reports were also examined to identify additional studies not captured in the initial database search.

**Figure 1:**
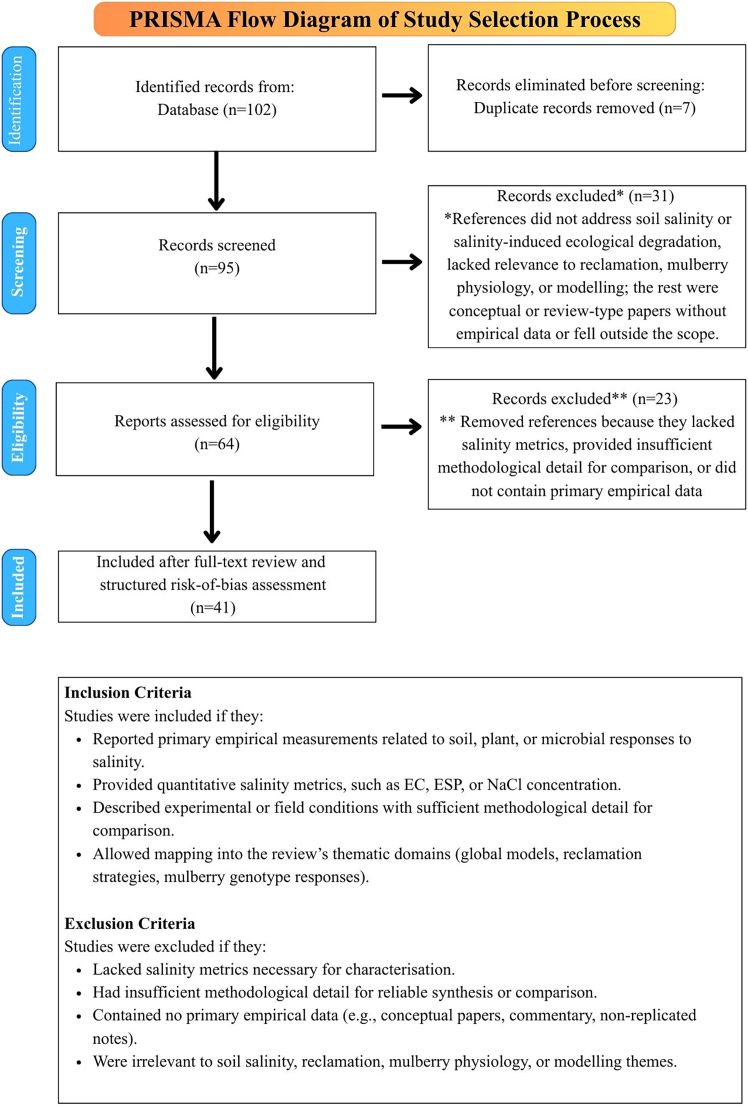
PRISMA flow diagram illustrating the identification, screening, and inclusion process used to select studies for the systematic review

The search process identified 102 records, from which 7 duplicate entries were removed, leaving 95 studies for title and abstract screening. After this stage, 64 studies were retained for full-text assessment, and 41 studies providing primary experimental data on soil, plant, or microbial responses to salinity were included in the final synthesis. Studies were included when salinity conditions were clearly characterized using measurable indicators such as EC, NaCl concentration, sodium adsorption ratio (SAR), exchangeable sodium percentage (ESP), or soil organic carbon (SOC). Review articles, conceptual papers, book chapters, and studies lacking explicit salinity measurements or methodological clarity were excluded. The overall study selection procedure is illustrated in Figure 1 (PRISMA flow diagram).

For each selected study, information was extracted on soil physicochemical properties (EC, pH, ESP, SAR, SOC, and soil texture), plant physiological responses (biomass production, chlorophyll content, K^+^/Na^+^ balance, proline accumulation, and antioxidant enzyme activity), microbial indicators, and reclamation interventions such as gypsum application, drainage management, halophyte utilization, and mulberry cultivation. Literature screening and data extraction were conducted using structured spreadsheets and manual verification to ensure consistent recording of variables across studies. Extracted information was synthesized using comparative qualitative analysis and thematic integration of evidence, recognizing the diversity of experimental designs, environmental conditions, and salinity levels reported across the reviewed studies. Included studies were categorized into four thematic groups: plant physiological tolerance, soil physicochemical restoration, field validation studies, and microbial or rhizosphere research, to assess the distribution of research emphasis. To enhance methodological reliability, the quality of included studies was evaluated during full-text screening based on clarity of experimental design, consistency of salinity measurement methods (e.g., EC, SAR, or ESP), and completeness of reported physiological or soil-response variables. Studies lacking clearly defined salinity characterization, reproducible experimental conditions or sufficient methodological transparency were excluded from the synthesis. This approach helped reduce potential reporting bias and ensured that the final dataset was derived from empirically robust and comparable studies.

An AI-enabled decision-support system incorporating the DNN was implemented using a harmonized dataset, integrating soil physicochemical, hydrological, and genotype-specific indicators. Model training, validation and performance evaluation procedures are described in the results and discussion section. While the present implementation demonstrates predictive feasibility within the available dataset, broader optimization across expanded datasets and independent regional validation remain necessary for large-scale operational deployment.

## Results and discussion

**Figure 2:**
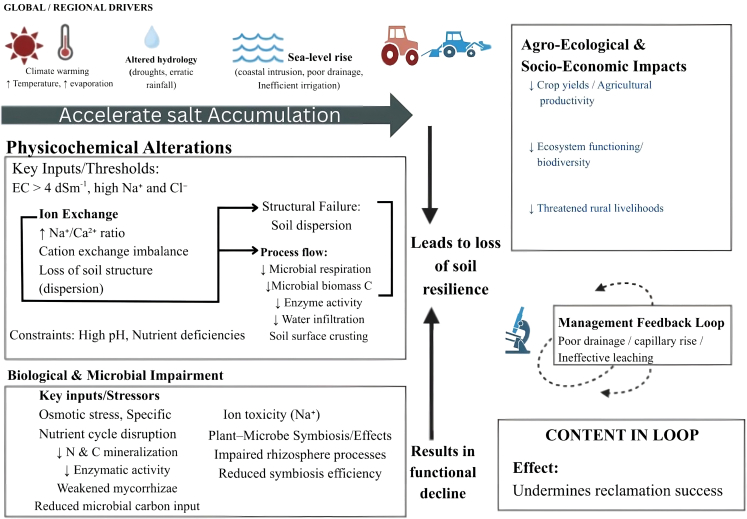
Conceptual framework showing how climatic and hydrological drivers accelerate salt accumulation and trigger physicochemical (Na^+^/Ca^2+^ imbalance, soil dispersion) and microbial degradation, leading to loss of soil resilience Feedback loop driven by poor drainage and ineffective leaching reinforces salinity persistence. The framework highlights practical management intervention points such as hydrological correction and soil-structure stabilization rather than isolated chemical amendments.

The synthesis of studies demonstrates that salinity functions as a coupled hydro-geochemical and biological stressor that progressively degrades soil structure, ion balance and ecosystem functioning. The interacting drivers and feedback loops underlying salinity-induced land degradation are conceptualized in Figure 2. Soil salinization results in osmotic stress and ion toxicity driven primarily by elevated Na^+^ and Cl^−^ concentrations. Excess Na^+^ displaces calcium (Ca^2+^) and magnesium (Mg^2+^) from exchange sites, destabilizing soil aggregates and reducing hydraulic conductivity.^4^^,^^5^ These physicochemical changes restrict leaching and reinforce salt accumulation, particularly in coastal and semi-arid environments.

Across the studies included in the PRISMA-guided synthesis, approximately 36% focused primarily on plant physiological and biochemical tolerance mechanisms, including ion regulation, antioxidant responses and genotype screening. Soil physicochemical restoration processes represented the largest thematic category, accounting for approximately 30% of the included studies. These investigations primarily evaluated key soil quality indicators, including EC, ESP, SOC, SAR, and soil aggregate stability, which are commonly used to assess salinity-induced soil degradation and recovery. Field-based or multi-season validation studies comprised roughly 20% of the analyzed literature, providing empirical evidence on the effectiveness of salinity mitigation strategies under realistic environmental conditions. In comparison, microbial- and rhizosphere-focused studies accounted for approximately 12%, reflecting the limited but growing attention to plant-microbe interactions in saline environments. The remaining studies focused on plant physiological responses, halophyte-based reclamation strategies, and agronomic management practices, highlighting the multidisciplinary nature of current research aimed at improving crop resilience and restoring salt-affected soils. This distribution highlights a strong experimental foundation in controlled physiological studies but comparatively limited long-term, landscape-scale validation, underscoring the need for expanded multi-location field trials and integrated datasets.

Biological processes decline concurrently under increasing salinity stress, particularly microbial biomass, and enzymatic activity.^8^^,^^29^ Microbial biomass carbon, respiration and enzyme activities are consistently suppressed under increasing salinity.^8^^,^^9^^,^^30^ Disruption of rhizosphere interactions weakens nutrient cycling and reduces soil resilience to hydrological variability.^31^^,^^32^ Climate change further intensifies these processes by increasing evapotranspiration and limiting natural leaching.^33^^,^^34^ These interacting mechanisms establish the ecological constraints within which restoration strategies must operate. A structured comparison of physicochemical, biological, and hybrid reclamation paradigms is presented in Table 1, providing a framework for interpreting restoration strategies across salinity-affected landscapes.

**Table 1:** Comparative evaluation of salinity-reclamation approaches with criterion-specific references

Evaluation Criterion	Physicochemical & Engineering Approaches	Biological & Bioengineering Approaches	Integrated Hybrid Approaches	References
Primary objective	rapid chemical correction of salinity through Na^+^ displacement and leaching.	ecological restoration using plant–microbe systems for sustainable amelioration.	combine rapid chemical correction with long-term ecological resilience.	Guangming et al.^35^
Dominant mechanisms	cation exchange (Ca^2+^ substitution), leaching, drainage-mediated hydrological flushing.	phytoremediation (halophytes), PGPR-mediated bioamelioration, SOM-driven nutrient cycling.	synergy of amendments, drainage, organic matter and salt-tolerant vegetation.	Thakur et al.^26^; Li et al.^36^; Jaiswal et al.^37^; Thakur et al.^38^; Kumar et al.^39^
Key inputs/amendments	gypsum, pyrite, lime, FGD gypsum, biochar.	halophytes, PGPR, microbial consortia, FYM, crop residues.	chemical amendments alongside organic matter, salt-tolerant species and drainage.	Ondrasek^33^; Li et al.^36^; Jaiswal et al.^37^
Effects on soil structure and function	rapid improvement: flocculation, aeration, hydraulic conductivity.	increase in SOM, microbial biomass, enzyme activity, nutrient cycling.	restoration of aggregation, cation balance and hydrological stability.	Singh et al.^40^
Recovery speed	fastest visible improvement under adequate leaching/drainage.	progressive, ecologically stable improvement.	efficient in short-term recovery + long-term sustainability.	Guangming et al.^35^; Li et al.^36^
Initial cost	high due to amendment quantity, drainage systems and technical labor.	low–moderate; compatible with smallholders.	moderate; varies with component blending.	Ondrasek^33^
Carbon footprint	Higher due to heavy amendments and engineering operations.	Low; increases soil carbon via biomass and microbial processes.	Lower than engineering-only + enhanced vegetation-driven carbon inputs.	Li et al.^36^; Jaiswal et al.^37^
Scalability	Scales via government-backed irrigation and drainage infrastructures.	Scales via community-led ecological restoration, low-input systems.	Scales across both engineered and community-driven frameworks.	Thakur et al.^38^; Kumar et al.^39^
Key limitations	Requires reliable freshwater for leaching; skilled implementation; costly drainage maintenance.	Requires consistent availability of quality inoculants and organic inputs; performance varies by region.	Requires institutional coordination, integrated monitoring and strong extension services.	Ondrasek^33^; Li et al.^36^

Source: Compiled by the authors.

**Figure 3:**
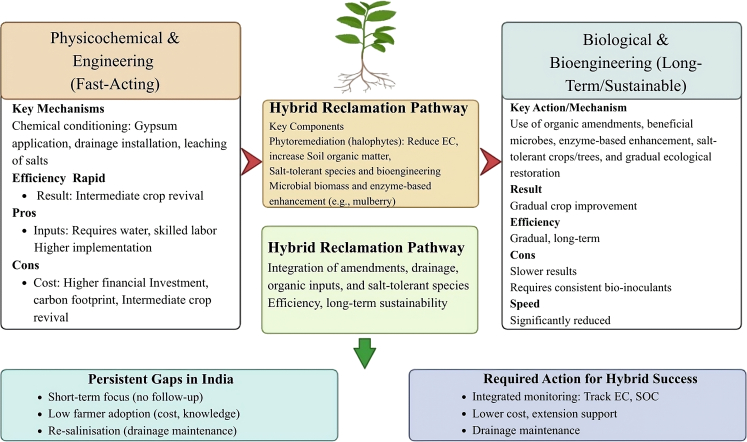
Comparative framework illustrating the major reclamation approaches used in salinity-affected landscapes, including physicochemical amendments, engineering solutions and biological restoration strategies The diagram highlights their mechanisms, relative effectiveness and long-term sustainability, emphasizing that integrated management combining targeted soil amendments, improved water management, and salt-tolerant vegetation provides a more practical and scalable pathway for restoring saline soils.

International restoration models indicate that durable salinity management requires integration of vegetative stabilization with long-term hydrological oversight and governance integration.^41^^,^^42^^,^^43^^,^^44^^,^^45^ A conceptual hybrid reclamation framework integrating physicochemical and biological strategies is presented in Figure 3. Halophyte-assisted systems in Spain and China improve soil structure and organic matter through phased ecological succession and irrigation management.^41^^,^^46^^,^^47^ Basin-scale monitoring frameworks in Australia and adaptive water governance in the Netherlands underscore the importance of coordinated drainage and long-term oversight.^27^^,^^42^

In contrast to coastal saline systems driven largely by marine intrusion and tidal hydrodynamics, inland saline landscapes such as western Rajasthan, Haryana canal-command regions and parts of the Indo-Gangetic plains are predominantly shaped by secondary salinization associated with shallow saline groundwater, inadequate drainage, and irrigation mismanagement.^48^ These systems often exhibit elevated ESP, sodium-induced aggregate dispersion and restricted vertical leaching, with seasonal EC fluctuations linked to irrigation intensity and evapotranspiration.^49^^,^^50^ Unlike chloride-dominant coastal salinity, inland salt-affected soils often contain sodium carbonate or bicarbonate salts that degrade soil structure and permeability.^3^^,^^15^

Under inland saline conditions characterized by elevated exchangeable Na^+^, restricted leaching and seasonal groundwater fluctuations, effective mulberry deployment must be integrated with drainage improvement, regulated irrigation and organic matter enhancement to stabilize soil structure and moderate sodium-induced dispersion. When hydraulic conductivity is partially restored, the perennial deep-root architecture of mulberry can further promote porosity development, gradual salt redistribution, and structural stabilization.^23^^,^^51^ Nevertheless, robust multi-location field validation across inland saline systems remains essential to confirm genotype performance under variable groundwater regimes and heterogeneous soil chemistry. Within this agro-ecological framework, mulberry emerges as a multifunctional bio-reclamation candidate.^17^^,^^18^^,^^19^^,^^20^ As NbS, it addresses salinity-driven land degradation through intrinsic ecosystem processes, such as ion regulation, rhizosphere-mediated aggregation and carbon inputs, while simultaneously generating socio-economic co-benefits through sericulture-linked livelihood systems.^19^ A comparative synthesis of international salinity-management strategies, including the proposed mulberry-based model, is presented in Table 2.

**Table 2:** International strategies for managing salinity-affected landscapes: a comparative matrix

Criteria	China: Halophyte Agroforestry & Drip Irrigation	Australia: Salinity Targets & Basin Governance	Central Asia: Integrated Soil & Salt-Tolerant Crop Management	Spain: Halophyte-Based Ecological Restoration	Netherlands: Nature-Based & Socio-Economic Adaptation	References	India: Proposed integrated Mulberry-Based Reclamation Model
Primary objective	convert saline soils into productive landscapes using drip-irrigated halophytes (e.g., *Suaeda salsa*) supporting food–fodder–bioenergy value chains.	maintain basin-wide salinity targets through interception schemes, governance tools and optimized irrigation to enhance ecological resilience.	reduce soil salinity and stabilize risks using mulching, terracing, efficient irrigation and salt-tolerant crops, including mulberry.	restore saline soils through native halophytes and natural succession.	mitigate coastal and inland salinity using ecological engineering and governance mechanisms.	Van Den Burg et al.^27^; Wang et al.^41^; Hart et al.^42^; Kulmatov et al.^43^; Zhang et al.^44^; Jiménez-Ballesta et al.^45^; Liang and Shi^47^; Wang et al.^52^; Guo et al.^53^; Suganuma et al.^54^; Koriyev et al.^55^	improve mulberry yield and soil quality in saline environments through hybrid ecological–hydrological management.
Strategy type	halophyte-based agroforestry, drip irrigation, intercropping and biological engineering.	deep-rooted revegetation, salt-interception schemes, modern irrigation and basin-level salinity credit–debit governance.	mulching, terracing, water–salt modeling and salt-tolerant cultivars.	active and passive halophyte succession using pioneer species.	salt marsh restoration, managed realignment, brackish agriculture and adaptive water governance.	Wang et al.^41^; Liang and Shi^47^; Wang et al.^52^; Koriyev et al.^55^; Peck and Hatton^56^	halophyte-led soil conditioning combined with efficient irrigation and salt-tolerant mulberry genotypes.
Restoration process	drip-irrigated halophytes reduce salinity, improve soil structure; intercropping accelerates desalinization.	interception schemes regulate salinity inflow; revegetation controls recharge for basin salinity compliance.	mulching reduces evaporative salt rise; terracing stabilizes runoff; modeling optimizes irrigation under uncertainty.	successional pathway: saline crust followed by pioneer halophytes then stable halophytic steppe.	salt marsh expansion buffers salinity and increases coastal resilience.	Liang and Shi^47^; Wang et al.^52^; Koriyev et al.^55^; Peck and Hatton^56^; Wang et al.^57^	soil preconditioning with halophytes, followed by mulberry establishment, then community-supported management.
Socio-economic context	low-resource, scalable models suited for rural arid zones; supports food–fodder–bioenergy chains.	strong basin governance; high initial cost but long-term gains for agriculture and ecosystems.	moderate cost; dependent on irrigation governance; key for rural and sericulture-linked livelihoods.	low-input restoration enhances ecosystem value in rural communities.	strong institutional capacity enables economic diversification under saline agriculture.	Hart et al.^42^; Kulmatov et al.^43^; Guo et al.^53^; Wang et al.^57^	moderate cost; supports sericulture-based rural livelihood systems.
Environmental advantages	improves soil fertility, biodiversity, SOM and long-term landscape resilience.	controls salinity, stabilizes groundwater, improves water quality and strengthens ecological function.	enhances soil moisture retention, yield stability and long-term soil health.	boosts biodiversity, improves soil structure and strengthens ecosystem stability.	enhances coastal defense, buffers salinity, improves water quality and increases agricultural resilience.	Van Den Burg et al.^27^; Wang et al.^41^; Jiménez-Ballesta et al.^45^; Liang and Shi^47^; Koriyev et al.^55^; Peck and Hatton^56^	improves soil structure, boosts microbial activity and increases mulberry productivity on saline soils.
Limitations	slower recovery under extreme salinity; reliant on local halophyte species.	high operational cost; requires continuous monitoring; vulnerable to climate variability.	irrigation mismanagement or excessive fertilizer use increases salinity risk.	slow recovery in highly saline soils; dependent on native halophyte diversity.	high investment needs; requires strong institutions and technical oversight.	Van Den Burg et al.^27^; Hart et al.^42^; Kulmatov et al.^43^; Jiménez-Ballesta et al.^45^; Wang et al.^57^	requires skilled irrigation management, capacity building and sustained farmer participation.
Relevance to mulberry cultivation	conditions soils for later mulberry introduction via halophyte-assisted desalinization.	governance-based salinity control frameworks can support mulberry systems in saline basins.	soil improvement and modeling approaches directly strengthen mulberry cultivation under salinity.	prepares degraded saline land for future mulberry agroforestry.	demonstrates integrated agriculture–governance approaches relevant to mulberry expansion.	Zhang et al.^44^; Guo et al.^53^; Wang et al.^57^	directly applies ecological and governance-based restoration to enable productive mulberry cultivation.

Source: Compiled by the authors

**Figure 4:**
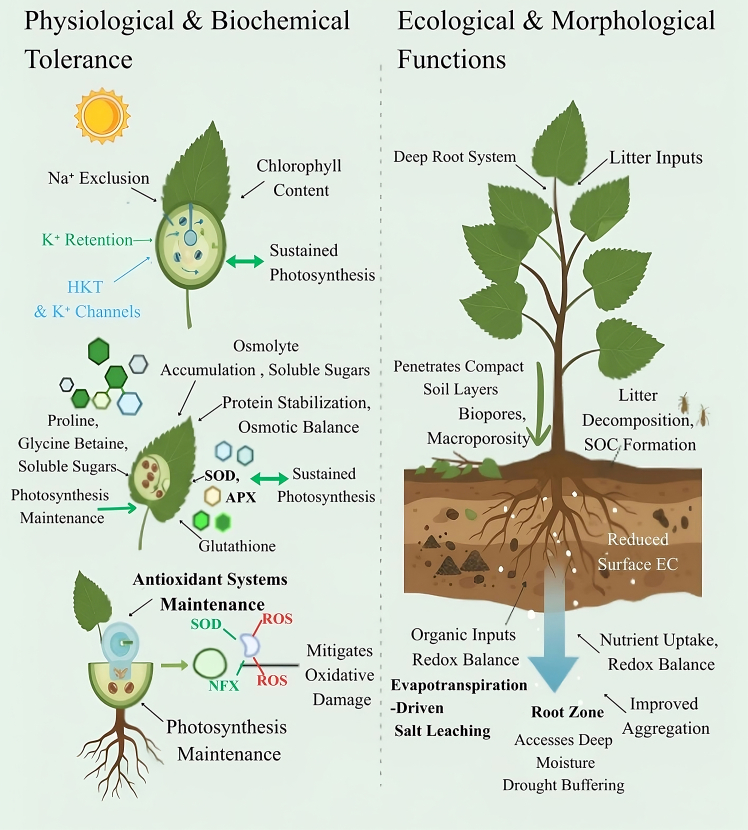
Integrated physiological and ecological mechanisms enabling mulberry (*Morus* spp.) to tolerate salinity and support soil recovery Key processes include Na^+^ exclusion, K^+^ retention, osmolyte accumulation, antioxidant defense, deep rooting, and litter-driven improvements in soil structure and carbon. These processes support the practical deployment of mulberry as a nature-based bio-reclamation plant species capable of improving soil conditions while sustaining agroforestry and sericulture-based livelihoods in salinity-affected landscapes.

Experimental and field-based evaluations have shown that selected mulberry genotypes exhibit tolerance to salinity levels reaching 19–23 dS m^−1^^18^^,^^19^^,^^20^ The integrated physiological, biochemical, and ecological mechanisms underpinning mulberry-based bio-reclamation are illustrated in Figure 4. Tolerance mechanisms include selective Na^+^ exclusion and maintenance of high K^+^/Na^+^ ratios mediated by high-affinity potassium transport systems.^22^ Osmotic adjustment through proline accumulation stabilizes cellular structures,^18^ while antioxidant enzymes such as superoxide dismutase, catalase, and ascorbate peroxidase mitigate oxidative stress.^20^^,^^21^ Genotypes including English Black, C776, and Kolitha-3 consistently demonstrate superior tolerance under saline conditions.^17^^,^^19^^,^^20^ A comprehensive chronological synthesis of mulberry salinity-tolerance studies, including *in vitro*, *ex vitro*, field and transgenic evaluations, is presented in Table 3. To translate genotype-specific tolerance evidence into operational deployment criteria, the synthesized findings were structured into EC-based salinity thresholds linked with management requirements (Table 4).

**Table 3:** Comprehensive chronological synthesis of mulberry (*Morus* spp.) salinity-tolerance studies

Exp. No.	Genotypes Examined	No. of Genotypes	Salinity Stressor	Study Type	Parameters Assessed	Major Findings	References
1	S1, ATP	2	NaCl	*Ex vitro*	physiological; biochemical	S1 exhibited stronger salt tolerance than ATP.	Kumar et al.^58^
2	S34, G2, G3, G4	4	NaCl	*In vitro*	morphological; physiological	G4 was identified as the most tolerant genotype.	Tewary et al.^59^
3	63 accessions (names not reported individually)	63	NaCl	*In vitro*	morphological; physiological	seven tolerant accessions selected.	Vijayan et al.^17^
4	English Black (♀), Rotundiloba (♀), Kolitha-3 (♀), C776 (♂), BC259 (♂), Tollygunj (♀), Mandalaya (♂)	7	NaCl	*Ex vitro*	morphological	English Black, Rotundiloba and C776 performed best.	Vijayan et al.^17^
5	English Black, Rotundiloba, Kolitha-3, Kolitha-7, Kolitha-8, Kolitha-9, Tollygunj, KPG-3, Tistavalley, Bushmald-B, Bishnupur-9, Bishnupur-4, Didia White, Dudhia Red, Mirgunj, Illoys, Assambola, MIX, CSRS-1, Berhampore-39, Berhampore-20, Berhampore-B, Berhampore-A, Kajli, Bogura-4, Philippine, Chinablack, Surat, Mysore Local, Punjab Local, Nagaland Local, Calabrase, China White, Shrim-8, OPH-1, Sujanpur-5, OPH-3, MNR, MS-5, Kanava-2, Takda, Sultanpur, Sujanpur	43	NaCl	*In vitro*	morphological; physiological	seeds of English Black, Rotundiloba, Kolitha-3, Mysore Local, Sultanpur & KPG-3 showed stable tolerance.	Vijayan et al.^60^
6	English Black, Rotundiloba, Mandalaya, Tollygunj, C776	5	NaCl	*Ex vitro*	morphological; anatomical; biochemical	tolerant genotypes showed superior physiological resilience.	Vijayan et al.^18^
7	SR1, SR2, SR3 (controlled breeding lines)	3	NaCl	*Ex vitro*	morphological; biochemical	SR3 showed superior tolerance.	Vijayan^61^
8	English Black, Rotundiloba, Mandalaya, Tollygunj, C776, and six derived hybrids	11	NaCl	*Ex vitro*	morphological; biochemical; physiological	recommended refinement of selection criteria for saline soils.	Vijayan^62^
9	K2 (transgenic lines)	1	NaCl	*Ex vitro*	morphological; biochemical; physiological; pathological; molecular	stress-inducible promoter improved tolerance without affecting silkworm feeding.	Das et al.^63^
10	K2, DD, RFS-175, S-13, C176, Ghosherami, AR12, S34, S1, C776	10	NaCl	*In vitro*	biochemical; physiological; molecular	S-34 and K2 were identified as strongly salt-tolerant.	Das et al.^64^
11	*Morus alba* (local), Sujanpuri	2	NaHSO_4_	*Ex vitro*	biochemical; physiological	local cultivar outperformed Sujanpuri.	Ahmad and Sharma^65^
12	ST02, ST17, ST25, ST33, ST46 (transgenic lines)	5	NaCl	*In vitro & Ex vitro*	morphological; biochemical; physiological; molecular	all transgenic lines exhibited higher salinity tolerance.	Checker et al.^66^
13	Surat, Michalal Farm, *M. indica* Black, Matigara White, UP-3, UP-16, TR-1, C-1726, C-1724, C-1748, ERRC-56, Chongpatgiri, Guhanathpuram, ERRC-170, ERRC-12, L-3, Sarnath-2 (17 genotype names identified from 20 accessions reported in the original study)	20	NaCl	*Ex vitro*	morphological; physiological; biochemical	large genotype-wise variability observed.	Jhansilakshmi et al.^67^
14	English Black, C776, BC_2_59, S1, Kolitha-3, Rotundiloba	6	environmental	field	biochemical; physiological	English Black, C776 & Kolitha-3 were identified as highly salt-tolerant and suitable for the coastal saline soils of South 24 Parganas, India.	Acharya et al.^20^
15	English Black, C776, Rotundiloba, BC_2_59, S1, Kolitha-3, Mandalaya, Assambola, C1690, White Badana, Behrampore-B, Thailand Lobed, Sultanpur, Kolitha-8, Sujanpur-5, Matigara Black, Kajli, Tollygunj, Dudhia White, China White, Mysore Local, China Black, Bush Malda-B, Punjab Local, Kosen	25	NaCl, environmental	*In vitro* and field	morphological; biochemical	English Black, C776 & Kolitha-3 confirmed as highly tolerant and suitable for coastal saline soils of South 24 Parganas, India.	Acharya et al.^19^

Source: compiled by the authors.

**Table 4:** Operational salinity thresholds for mulberry-based bio-reclamation across EC gradients

EC Range (dS m^−1^)	Salinity Category	Expected Plant Response	Recommended Genotypes	Management Requirements	Reference	Reclamation Objective
< 4	non-saline	normal growth	all commercial cultivars	standard agronomy	Datta^68^	conventional production
4–8	mild salinity	minimal stress; slight physiological adjustment	English Black, C776, Kolitha-3, Rotundiloba, BC_2_59, S1, Mandalaya, Punjab Local, Thailand Lobed, China White, China Black	organic amendments; irrigation monitoring	Vijayan et al.^17^; Acharya et al.^19^	productivity and preventive reclamation
8–15	moderate salinity	reduced growth; strong ion regulation required	English Black, C776, Kolitha-3, Rotundiloba, BC_2_59, S1	drainage support; organic matter addition; raised beds	Vijayan et al.^17^; Vijayan et al.^18^; Acharya et al.^19^; Acharya et al.^20^	active bio-reclamation
15–23	high salinity	genotype-dependent survival; reduced biomass	English Black, C776, Kolitha-3	integrated drainage, organic inputs and careful irrigation scheduling	Vijayan et al.^17^; Acharya et al.^19^; Acharya et al.^20^	stabilization and gradual soil recovery
> 23	severe salinity	high mortality risk	no recommended genotypes yet	requires engineering intervention before planting	Acharya et al.^19^; Acharya et al.^20^	not recommended for direct mulberry deployment

Source: Compiled by the authors.

Mulberry further contributes to soil ecological recovery through its perennial, robust root system, which is effective for soil and water conservation,^23^ and, like other vegetation, mulberry contributes to improved soil structure by enhancing soil porosity and water infiltration through increased aggregation and pore development.^51^ Litter inputs promote SOC accumulation and microbial regeneration,^69^ while agroforestry systems incorporating mulberry enhance carbon sequestration in degraded landscapes.^51^ Unlike purely amendment-based reclamation approaches that chemically replace exchangeable Na^+^,^70^ mulberry can promote progressive biological stabilization through sustained plant-soil interactions.^23^

A distinguishing advantage of mulberry-based bio-reclamation lies in its integration with sericulture. As a major rural employment sector engaging millions, sericulture embeds ecological restoration within livelihood systems.^24^^,^^25^ Globally, mulberry-based sericulture systems are most prominent in China and India, but also contribute to rural economies in several Asian and Mediterranean countries, reinforcing the transnational relevance of mulberry-driven restoration models.^71^^,^^72^ This alignment of environmental recovery with socio-economic resilience strengthens mulberry’s long-term adoption potential and differentiates it from halophytes, which are primarily valued for ecological tolerance.

### Comparative economic feasibility of mulberry-based bio-reclamation system

Beyond ecological performance, the scalability of salinity-reclamation systems depends critically on their relative economic viability compared with alternative salt-tolerant crops and physicochemical reclamation strategies.^14^^,^^15^^,^^28^ Conventional amendment-based approaches in India, particularly gypsum application combined with subsurface drainage, can reduce ESP and improve soil permeability under favorable hydrological conditions.^11^^,^^70^ However, these interventions are capital-intensive, require repeated input application, and depend on reliable water availability and institutional support.^12^ Long-term maintenance costs and drainage infrastructure further increase financial barriers for smallholders in canal-command and coastal systems, limiting the adoption of conventional engineering-based reclamation approaches.^73^

In contrast, vegetative and agroforestry-based reclamation strategies generate progressive ecological recovery while offering varying levels of economic return. Species such as *Prosopis juliflora*, *Casuarina equisetifolia*, and other salt-tolerant trees improve soil structure and tolerate elevated salinity, but their economic return is often limited to timber, fuelwood or long-rotation biomass production.^14^^,^^15^ Halophyte grasses and salt-tolerant fodder systems may stabilize soils under high salinity, yet typically provide narrow livelihood pathways without strong integration into high-value markets.^15^^,^^28^

Mulberry-based bio-reclamation differs structurally because ecological restoration is directly embedded within an established sericulture value chain. India’s sericulture sector engages millions of rural households and generates over 500 employment days per acre annually.^24^^,^^25^ These systems demonstrate the transnational relevance of mulberry-based agroforestry models.^74^ Unlike one-time amendment costs, mulberry plantations provide recurrent leaf harvests that support sustained silk production and income generation.^75^ Furthermore, mulberry’s perennial root architecture enhances soil aggregation and organic carbon inputs,^23^ contributing ecosystem-service value that amendment-only systems do not inherently provide. Agroforestry integrations incorporating mulberry have also demonstrated improved carbon storage and soil-quality enhancement in degraded landscapes.^51^

Although detailed farm-level cost–benefit datasets across agro-ecological zones remain limited, the available comparative evidence indicates that mulberry-based bio-reclamation offers a favorable balance between moderate establishment cost, sustained annual returns, employment generation and long-term soil improvement relative to gypsum-dominated systems and non-market-oriented halophyte plantations. Under moderate to high salinity conditions where conventional cropping becomes economically unstable, mulberry thus represents a comparatively resilient techno-ecological alternative.

### AI-enabled decision-support system

To translate the synthesized physiological and soil-response evidence into operational planning support, an AI-enabled decision-support system was developed to predict mulberry salinity tolerance using integrated soil, hydrological and genotype-specific indicators derived from harmonized field and experimental observations. The modeling dataset (*n* = 50) consisted of harmonized field and experimental observations extracted from studies reporting comparable salinity-response variables and genotype performance data. Each observation included predictor variables such as EC (dS m^−1^), SAR, ESP (ESP; represented in the model as Exchangeable_Na_pct), soil pH, organic matter content (%), chloride concentration (%), soil depth (cm), irrigation intensity, groundwater influence and genotype identity (e.g., Kolitha-3 and other screened cultivars). Multiple observations were derived from individual studies where different salinity treatments, soil conditions or genotypes were evaluated. The response variable was defined as a continuous tolerance score (0–10) representing relative genotype performance under graded salinity exposure, derived from reported physiological indicators including biomass retention, chlorophyll stability and maintenance of favorable K^+^/Na^+^ ratios. All variables were standardized to common units to ensure cross-study comparability prior to model development. The compiled dataset used for model training and validation is summarized in Table S1.

**Figure 5:**
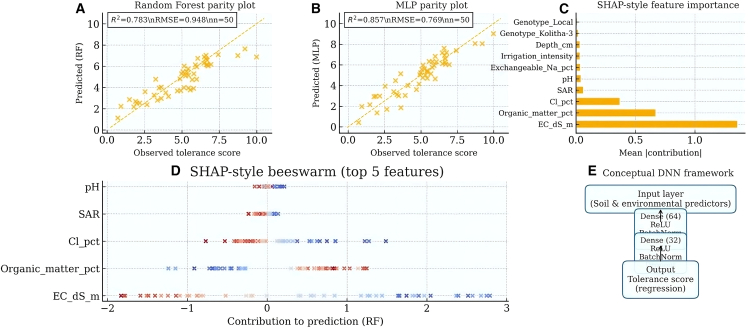
AI-enabled decision-support modeling for mulberry salinity suitability assessment (A) Random forest parity plot and (B) MLP parity plot showing observed versus predicted tolerance scores with test-set performance metrics (R^2^, RMSE; *n* = 50), demonstrating stronger nonlinear predictive capacity of the DNN. (C) SHAP-style feature-importance analysis identifying EC as the dominant predictor, followed by organic matter, chloride concentration, and SAR. (D) SHAP beeswarm plot illustrating direction and magnitude of predictor contributions across samples. (E) Implemented DNN system showing input features, two hidden layers (64→32 units) with ReLU, batch-normalization, and dropout and a linear output for tolerance prediction.

Two algorithms were evaluated: random forest (RF) and a deep multi-layer perceptron (MLP) model. Data were normalized before modeling and partitioned using a 70:30 train-test split, with 70% of observations used for model training and 30% reserved for independent performance evaluation. Model performance and feature attribution results are presented in Figure 5. To enhance robustness given the moderate dataset size, 5-fold cross-validation was implemented during training, and performance metrics were averaged across folds to assess stability and reduce sampling bias.

Predictive accuracy was quantified using the coefficient of determination (R^2^) and root-mean-square error (RMSE). The deep MLP model demonstrated superior predictive performance (test R^2^ = 0.857; RMSE = 0.769) compared with the RF model (test R^2^ = 0.783; RMSE = 0.948), indicating improved capture of nonlinear interactions among salinity intensity, ion composition, and genotype-specific traits. The consistency between cross-validated and independent test performance suggests limited overfitting within the available data range. Detailed cross-validation results and performance metrics across folds are provided in Table S2.

Parity plots indicate the absence of systematic over- or under-prediction across the tolerance gradient, with residual dispersion remaining relatively consistent along the observed range.

Model interpretability was examined using shapley additive explanations (SHAP)-based feature attribution analysis. EC emerged as the dominant predictor, followed by organic matter content, chloride concentration, SAR and soil pH. Mean SHAP contribution values for all predictors are summarized in Table S3. This ranking is mechanistically consistent with established salt-stress pathways: EC reflects osmotic stress intensity; SAR and exchangeable Na^+^ influence structural degradation and ionic imbalance; organic matter moderates aggregation, infiltration, and buffering capacity. Directional SHAP contributions showed that elevated SAR and chloride shifted predictions negatively, whereas higher organic matter improved predicted tolerance under moderate EC conditions. The alignment between statistical attribution and ecophysiological understanding strengthens model credibility. In addition to feature-level interpretability, observed-predicted parity analysis further demonstrated strong agreement across the tolerance gradient without systematic bias, reinforcing overall model reliability (Table S4).

The implemented DNN system consisted of an input layer integrating soil and environmental predictors; two dense hidden layers (64 and 32 neurons) with rectified linear unit (ReLU) activation and batch normalization; and a regression output layer generating a continuous tolerance score.

For land-management agencies, the decision-support system requires three primary data inputs: (1) spatially resolved salinity indicators (EC, SAR, ESP, pH, and SOC) obtained from soil surveys or calibrated remote sensing; (2) irrigation and groundwater metrics reflecting hydrological pressure; and (3) genotype performance data across defined EC gradients. Model outputs may be expressed either as a continuous tolerance score or categorized into operational suitability classes aligned with established EC thresholds (for example, 4–8, 8–15, and 15–23 dS m^−1^). When integrated with regional salinity mapping, the system enables prioritized site selection, genotype matching and resource allocation for mulberry-based bio-reclamation initiatives.

Notwithstanding its predictive strength, the modeling system remains constrained by moderate sample size and geographic concentration of available data; the AI-enabled architecture should therefore be interpreted as a conceptual data-integration system that illustrates how heterogeneous ecological and physiological variables may be structured to support future salinity suitability assessment. Expanded multi-location datasets, independent regional validation, and long-term monitoring across inland and coastal saline systems are required to enhance generalizability and institutional deployment. Nevertheless, the present analysis demonstrates the feasibility of integrating soil salinity intensity, structural modifiers and genotype tolerance into a unified, quantitatively grounded decision-support system.

## Implementation

The findings suggest that mulberry-based bio-reclamation offers a biologically grounded strategy for integrating ecological restoration with national priorities in climate-adaptive agriculture and degraded land management. Salinity-affected regions in Gujarat, Odisha, West Bengal, and canal-command systems represent landscapes where bio-reclamation linked with livelihood systems may reduce long-term dependency on costly chemical amendments. Comparable saline-prone agro-ecological zones also occur in inland China and Central Asia, where similar perennial agroforestry integration may be feasible under regulated irrigation systems.

Compared with alternative salinity-tolerant crops such as barley or halophytes (e.g., *Salicornia* spp.),^76^^,^^77^ mulberry offers a comparatively established and vertically integrated value chain through sericulture, encompassing cocoon production, silk processing, and associated rural enterprises.^78^ While halophytes contribute primarily to ecological stabilization and niche markets, and species such as *Prosopis juliflora* are often limited to fuelwood use,^79^ mulberry directly links land restoration with sustained income generation and employment opportunities.^19^ This livelihood integration enhances long-term adoption potential and improves economic feasibility relative to reclamation models that rely solely on ecological performance without embedded market systems.

Gypsum-based reclamation of moderately saline soils requires repeated applications of 2–10 t ha^−1^ (depending on ESP) and drainage investments, leading to high upfront costs.^11^^,^^15^ In contrast, mulberry establishment involves standard perennial inputs but delivers sustained returns through sericulture, with irrigated leaf yields of 15–25 t ha^−1^ yr^−1^^80^ depending on genotype and salinity.^19^ Supported by established cocoon and silk value chains,^81^ mulberry reduces market-entry risk relative to niche halophytes such as *Salicornia* spp. Although site-specific profitability varies, integrating ecological restoration with an existing agro-industrial system like sericulture offers stronger long-term economic viability^74^ than amendment-based reclamation without embedded livelihood returns.

However, successful scaling requires integration with irrigation governance, drainage maintenance and institutional coordination. International evidence demonstrates that vegetative restoration succeeds only when embedded within hydrological regulation and monitoring systems.^27^^,^^42^ Without such support, biological gains risk reversal.

## Knowledge gaps

Despite substantial progress, critical knowledge gaps remain regarding scalability beyond coastal eastern India. Inland soil salinization often exhibits higher ESP and distinct hydrological dynamics compared with coastal saline systems, potentially influencing mulberry establishment and long-term soil feedback mechanisms. Comparative field trials across canal-command regions and inland saline belts are therefore necessary to determine ecological transferability.

Mechanistically, coastal soil salinization typically occurs by soluble salts derived from marine intrusion, where high EC primarily reflects osmotic stress and chloride toxicity. In contrast, inland saline systems exhibit structurally sodium-dominated profiles that may alter mulberry establishment dynamics relative to chloride-dominant coastal systems. These mechanistic differences underscore the importance of region-specific validation before large-scale deployment. Globally, where more than 1 billion hectares are affected by salinity, even modest integration of perennial salt-tolerant agroforestry species within moderately saline inland zones could represent a transformative restoration opportunity at the landscape scale.

Long-term ecosystem feedback processes remain insufficiently quantified. Multi-year monitoring of soil EC, ESP, and SOC trajectories is required to determine whether mulberry-based bio-reclamation induces sustained soil stabilization or requires complementary management interventions. Additionally, genotype × environment interactions across heterogeneous soil types remain poorly characterized, limiting predictive deployment across diverse agro-ecological zones.

## Future directions

Future research should prioritize multi-location, longitudinal field trials spanning at least 5–10 years to capture temporal salinity fluctuations and ecosystem feedback processes. Integration of soil microbiome profiling and metagenomic approaches would clarify restoration-driven shifts in microbial community structure. Quantitative assessment of carbon sequestration rates under mulberry-based bio-reclamation, expressed in Mg C ha^−1^ yr^−1^, is necessary to evaluate climate mitigation potential.

Furthermore, systematic evaluation of genotype × environment interactions across contrasting soil textures and hydrological regimes will strengthen ecological generalization. Coupling remote sensing datasets with ground-based salinity measurements may enhance spatial modeling accuracy and support scalable decision-support implementation.

## Conclusion

Soil salinity poses an increasing threat to ecological stability, agricultural productivity and rural livelihoods across arid and semi-arid regions worldwide, with particularly severe impacts in India. The evidence synthesized in this review demonstrates that mulberry-based bio-reclamation represents a multifunctional NbS that combines physiological salt tolerance, soil structural improvement, and livelihood generation through sericulture. Although expanded multi-location validation and harmonized datasets remain necessary, current evidence supports their deployment within defined ecological thresholds as a viable pathway for restoring varied levels of salinity-affected landscapes. Integration of biological resilience with structured environmental planning represents a deployable, threshold-defined and decision-support-enabled restoration system for saline landscapes, with obvious further necessity of long-term field evaluation.

### Limitations of the study

This review synthesized evidence derived from heterogeneous experimental, field and modeling studies conducted across diverse agro-ecological conditions. Although the PRISMA-guided framework improved methodological transparency, several limitations remain. First, substantial variability among included studies in terms of salinity metrics, experimental duration, genotype selection and management conditions constrained direct quantitative meta-analysis. Second, the reviewed evidence base remains unevenly distributed across agro-ecological regions, potentially limiting broader comparative interpretation across highly heterogeneous saline systems. Third, the AI-enabled decision-support framework was developed using a moderately harmonized dataset (*n* = 50), and independent validation with larger, multi-regional datasets remains necessary to improve predictive generalizability.

## Data and code availability

The produced data have been made available in the article and supplemental information.

## Acknowledgments

The authors gratefully acknowledge the support provided by the Hon’ble Principal of Hiralal Mazumdar Memorial College for Women, Dakshineswar, India and the Hon’ble Vice-Chancellor of Raiganj University, India, which made possible the completion of this review.

This research did not receive any specific grant from funding agencies in the public, commercial, or not-for-profit sectors.

## Author contributions

Supervision, writing – original draft, writing – review and editing, Visualization, validation, methodology, investigation, formal analysis, data curation, conceptualization, R.A.; supervision, validation, conceptualization, S.R.A. supervision, validation, resources, conceptualization, D.G.; investigation, methodology, S.S.; writing – original draft, validation, investigation, resources, conceptualization, A.M.; resources, S.D.; investigation, resources, S.C.; resources, methodology, investigation, Conceptualization, D.S.; methodology, investigation, R.C.

## Declaration of interests

The authors declare no competing interests.

## Declaration of generative AI and AI-assisted technologies in the writing process

During the preparation of this work the authors used ChatGPT 5.2, OpenAI in order to (1) draft explanatory text related to the machine-learning workflow, (2) generate RF, MLP, SHAP, and DNN analytical figures and (3) format supplemental materials. After using this tool, the authors reviewed and edited the content as needed and take full responsibility for the content of the published article.

## Supplemental information

Table S1. Harmonized Dataset Used for AI-Based Modelling of Mulberry Salinity Tolerance

Table S2. Cross-Validation Performance Metrics of Random Forest and Deep Neural Network Models

Table S3. SHAP-Based Feature Importance Rankings for Predictors of Mulberry Salinity Tolerance

Table S4. Observed–Predicted Performance Statistics and Residual Analysis of the AI-Based Salinity Tolerance Models
